# 

**Published:** 1876-09

**Authors:** 


### Table of Contents.

ORIGINAL COMMUNICATIONS.

Report of Sixty-Nine Cataract Operations. *By A. W. Calhoun, M.D., Atlanta, Ga* .... 321

ATLANTA ACADEMY OF MEDICINE.

Organic Urethral Stricture.... 347

OUR EXCHANGES.

The Air an Anæsthetic.... 354
Bed-Bug or no Bed-Bug.... 358
Salicylic Acid in Rheumatism.... 359
Herniotomy for Reducible Inguinal Hernia.... 360
The Fisher Case.... 361
Poison-Oak Eruption.... 363
Sulphate of Quinine as an Excitant of the Uterus....
Nerve-Stretching in Tetanus.... 364
The Treatment of Hypospadias.... 365
The Treatment of Ovarian Cysts by Drainage....
Treatment of Capillary Nævus.... 366
The Determination of Sex....
Treatment of Prolapsus Ani in Children.... 367
Raw Onions as an Hypnotic....
Brain Lesion.... 368
Glycerine as a Therapeutic Agent....
Transferance of Measles to Dogs....
Salicin and Salicylic Acid in Rheumatism.... 369
Differences between the Anæsthesia of Ether and Chloroform.... 370
The Value of Pressure in Semi-malignant Mammary Tumours.... 371
Pathognomonic Symptom of Cerebro-Spinal Meningitis.... 373
Hæmoptysis Treated by Ergot....

BIBLIOGRAPHICAL.

Cyclopædia of the Practice of Medicine.... 373
Medical and Surgical Memoirs.... 374
A Treatise on Therapeutics.... 376
Atlas of Skin Diseases....
Micro Photographs in Histology, Normal and Pathological.... 377
Pamphlets Received....

EDITORIAL.

Salicylic Acid.... 379
Child Crying in Utero.... 380
Transactions of the Medical Association of the State of Alabama.... 381
An Interesting Family.... 383
Transactions of the Texas State Medical Association.... 384

